# Pathogen-Induced Epigenetic Modifications in Cancers: Implications for Prevention, Detection and Treatment of Cancers in Africa

**DOI:** 10.3390/cancers13236051

**Published:** 2021-12-01

**Authors:** Alexandra Lindsey Djomkam Zune, Charles Ochieng’ Olwal, Kesego Tapela, Oloche Owoicho, Nora Nghochuzie Nganyewo, Frank Lyko, Lily Paemka

**Affiliations:** 1 West African Centre for Cell Biology of Infectious Pathogens (WACCBIP), Department of Biochemistry, Cell and Molecular Biology, College of Basic and Applied Sciences, University of Ghana, Accra LG 54, Ghana; coolwal@st.ug.edu.gh (C.O.O.); ktapela@st.ug.edu.gh (K.T.); oowoicho@st.ug.edu.gh (O.O.); nnnganyewo@st.ug.edu.gh (N.N.N.); 2 Department of Biological Sciences, Benue State University, Makurdi P.M.B. 102119, Benue State, Nigeria; 3 Medical Research Council Unit The Gambia at London School of Hygiene and Tropical Medicine, Banjul P.O. Box 273, The Gambia; 4 Division of Epigenetics, DKFZ-ZMBH Alliance, German Cancer Research Center, 69120 Heidelberg, Germany; f.lyko@dkfz-heidelberg.de

**Keywords:** epigenetics, tropical pathogens, cancer, pathogen-induced cancer

## Abstract

**Simple Summary:**

Cancer is a genetic disease; hence, pathogens are likely to cause cancers via genetic alterations, including epigenetic modification, a change in gene expression without changes in the DNA sequences. However, the mechanism(s) by which pathogens induce or enhance cancer development remains unclear. Studies have reported associations between some infectious pathogens and epigenetic changes, implying that pathogens could be involved in cancer development through the modification of host epigenetic factors. With the high burden of infectious pathogens, Africa is at elevated risk of pathogen-mediated cancers. A better understanding of the role of tropical infectious pathogens in regulating epigenetic modifications associated with cancer development could provide resources to tame the potential rise of pathogen-associated cancers in Africa. This review discusses cancer epigenetic studies in Africa, the link and potential mechanisms by which tropical pathogens induce carcinogenesis and opportunities as well as challenges for cancer management.

**Abstract:**

Cancer is a major public health burden worldwide. Tumor formation is caused by multiple intrinsic and extrinsic factors. Many reports have demonstrated a positive correlation between the burden of infectious pathogens and the occurrence of cancers. However, the mechanistic link between pathogens and cancer development remains largely unclear and is subject to active investigations. Apart from somatic mutations that have been widely linked with various cancers, an appreciable body of knowledge points to alterations of host epigenetic patterns as key triggers for cancer development. Several studies have associated various infectious pathogens with epigenetic modifications. It is therefore plausible to assume that pathogens induce carcinogenesis via alteration of normal host epigenetic patterns. Thus, Africa with its disproportionate burden of infectious pathogens is threatened by a dramatic increase in pathogen-mediated cancers. To curb the potential upsurge of such cancers, a better understanding of the role of tropical pathogens in cancer epigenetics could substantially provide resources to improve cancer management among Africans. Therefore, this review discusses cancer epigenetic studies in Africa and the link between tropical pathogens and cancer burden. In addition, we discuss the potential mechanisms by which pathogens induce cancers and the opportunities and challenges of tropical pathogen-induced epigenetic changes for cancer prevention, detection and management.

## 1. Introduction

Globally, an estimated 19.3 million new cancer cases and almost 10.0 million cancer deaths occurred in 2020. The global cancer burden is expected to reach 28.4 million cases in 2040, a 47% rise from 2020. The relative expected magnitude of increase is most striking in low Human Development Index countries (95%), especially in Africa [1]. Many of those cases are preventable or effectively treatable at early stages. However, achieving early cancer diagnosis and efficient treatment remain challenging owing to the multiple causalities of the disease and the high infrastructural costs [2] required for cancer diagnosis and management.

Infectious pathogens are a major cause of cancer, especially in low-and middle-income countries, which are ill- equipped to manage the disease. Therefore, the focus should be directed towards the prevention of infections, as this could reduce the development of pathogen-related cancers. It is estimated that in 2018, 2.2 million out of a total of 18 million new cancer cases worldwide were caused by infectious pathogens [3]. In low-income countries mostly in Africa, over 60% of diseases are attributed to infectious pathogens in contrast to high-income countries where less than 5% of diseases are linked to pathogens [4]. The proportion of infection-mediated cancers varies greatly by geographical region [5]. In Africa, 24.5% of cancers are attributed to infectious pathogens [6]. This may be an underestimation considering the limited cancer diagnosis infrastructure in most African countries. In contrast, less than 5% of cancers are attributed to infectious pathogens in high-income countries [5].

The involvement of infectious pathogens in carcinogenesis poses a serious threat to the fight against cancer, especially in the tropical regions, which have a disproportionately high burden of infectious pathogens. Africa harbors several tropical pathogens, including *Plasmodium* spp., *Leishmania*, *Schistosoma* spp., *Trypanosoma* spp., *Brugia* spp., Dengue virus, Chikungunya virus, *Wuchereria bancrofti* and *Onchocerca volvulus*. Generally, the etiological role of many pathogens in carcinogenesis remains unclear. Several studies have suggested that *Helicobacter pylori* and Human papillomavirus (HPV) involved in gastric cancer and cervical cancer, respectively, induce carcinogenesis by altering gene expression through epigenetic mechanisms (reviewed in [7]). Furthermore, some of the endemic tropical pathogens, such as *S. mansoni* [8], *O. volvulus* [9] and *P. falciparum* [10], induce inflammation, a key driver of carcinogenesis (reviewed in [11]). Due to their unique diets and lifestyle, Africans tend to have a distinct set of microbiomes [12]. The increasing evidence of microbiome-associated cancers via alteration of the local immune system, systemic deregulation and dysregulation of antitumor immunity [13] suggest that Africans may be at elevated risk of pathogen-associated cancers. Detailed studies aimed at understanding the precise role of tropical pathogens in cancer development could inform on cancer prevention, early diagnosis and pharmacogenomically aided therapy.

Cancer burden is directly proportional to the prevalence of infectious pathogens in Africa [3]. However, research studies on pathogen-induced epigenetic modifications in Africa remain scarce. Thus, in this review we highlight the cancer epigenetic studies and the link between tropical pathogens and cancer burden in Africa. Furthermore, we discuss the potential mechanisms by which pathogens induce cancers, as well as opportunities and challenges that tropical pathogen-induced epigenetic changes present in Africa.

## 2. Epigenetics in Cancer Development and Progression

Epigenetics is a term that describes reversible heritable changes in gene expression without variations in the DNA sequence [14]. Epigenetic deregulation has been recognized as a key player in carcinogenesis [15,16,17]. Observed epigenetic alterations include changes in DNA methylation, histone modifications and nucleosome positioning [18]. Changes in the epigenome disrupt gene regulation, resulting in cancer-promoting gene expression patterns. Known epigenetic mutations include genome-wide hypomethylation and regional hypermethylation, particularly in promoter-associated CpG islands [19,20,21], universal changes in histone modification marks [21,22] and deregulation of non-coding RNAs [21,23,24].

Adding to the growing knowledge on epigenetics and its role in cancer biology, several studies have demonstrated how deregulation in different epigenetic mechanisms is associated with cancer [25,26,27,28]. It was also demonstrated that the epigenetic alterations leading to carcinogenesis were caused by infectious pathogens; for instance, a proposed mechanism of gastric carcinogenesis by *H. pylori* is the silencing of miR-490-3p, which reactivates the Chromatin Remodeler SMARCD1 [29]. Moreover, higher methylation rates of *RARbeta2* and *APC* genes have been observed in Schistosoma-associated bladder cancer compared to non-Schistosoma bladder cancer patients [30].

DNA methylation changes, which involve both hypomethylation and hypermethylation, have received the most attention in epigenetic studies, as aberrations in this modification are common in virtually all cancers [31]. In cancerous cells, DNA hypermethylation has mostly been reported at promoter CpG islands of key genes involved in processes such as cell cycle regulation, DNA repair, apoptosis and differentiation [32]. Moreover, high-throughput analyses of genome-wide DNA methylation have demonstrated distinct epigenetic signatures that correlate with tumor stage and type are reproducibly found in nearly all cases of specific types of cancers [20,33].

Histone modification changes in cancers are less well understood compared to DNA methylation changes. Histone modification enzymes have been shown to exhibit distinct patterns of expression depending on the tissue of origin and can discriminate tumors from their matched normal tissues, clustering them according to cell type [34]. This indicates that abnormal expression of histone modification enzymes are involved in cancer-specific neoplastic transformation [34].

Histone mutations have recently been associated with various cancers. For example, mutations in histone H3 have been reported with high genetic penetrance in rare pediatric gliomas and sarcomas [35,36,37]. These histone mutations have led to the coining of the term “oncohistones”, with several mutations being associated with cancers. For instance, oncohistone H3K27M and H3G34V/R have been identified in diffuse intrinsic pontine gliomas and pediatric glioblastomas, respectively [35,36]. Other studies have shown that the histone H3 variant H3.3 is modified at lysine 36 (H3.3K36M) in 95% of chondroblastomas and at glycine 34 (H3.3G34W/L) in 92% of giant cell tumors of the bone [37]. Oncohistones have also been observed in diffuse large B-cell lymphomas (histone H1), head and neck cancers (H3K36M) and carcinosarcomas (H2A and H2B) [38,39,40]. In some cases, the histone mutations are found to be the only recurrent mutation identified in the tumor, suggesting that they are not only associated with cancer development but also act as initiators [35]. A distinct feature of these common oncohistone mutations is their occurrence at or near key regulatory post-translational modifications in the histone tails, implying a potential interference with the ‘reading’, ‘writing’ and/or ‘erasing’ of these regulatory marks [41].

**Figure 1:**
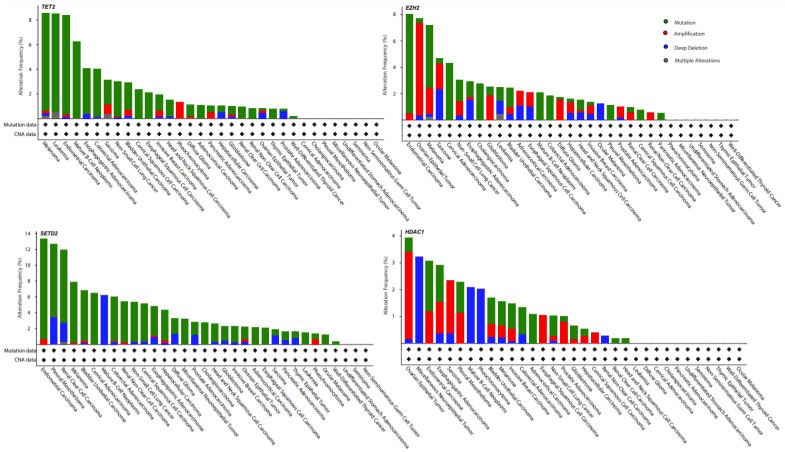
Genetic alteration in epigenetic regulating genes across various tumor types. TCGA Pan cancer data from 32 studies were analyzed on cBioPortal for Cancer Genomics. The bars represent the genetic alteration (mutations, amplifications, deep deletions, etc.) frequencies of *TET2*, *SETD2*, *EZH2* and *HDAC1* across different cancers. The analysis was performed as detailed previously [42,43].

The frequent genetic alterations in epigenome regulating genes suggest their centrality in carcinogenesis. As shown in Figure 1, our analysis of a few epigenetic regulating genes, such as *SETD2*, *HDAC1, TET2* and *EZH2*, using The Cancer Genome Atlas (TCGA) Pan Cancer dataset on cBioPortal for Cancer Genomics (https://www.cbioportal.org/, accessed on 8 November 2021) revealed that these regulatory genes are frequently altered in different cancer types. Thus providing supporting evidence implicating epigenetic regulation as a mechanism underlying tumor development and progression.

Non-coding RNAs play an integral role in gene expression regulation. Their deregulation as a result of amplifications, deletions and mutations as well as through other regulator genes have been associated with a growing number of cancers through aberrant functioning of their specific targets [44]. Non-coding RNAs can act as oncogenes or tumor-suppressors. In chronic lymphocytic leukemia (CLL) patients, deletion at the 13q14 region that encodes miR-15 and miR-16 is common [45]. The miR-15 and miR-16 miRNAs are implicated in apoptosis through their targeting of B-cell lymphoma 2 (*BCL*-2) [45]. Another example of non-coding epigenetic regulation is seen in B cell lymphoma, where the miR-17~92 cluster (13q31-q32) is amplified and acts with *MYC* to promote tumor development in hematopoietic malignancies [46].

## 3. Overview of Cancer Epigenetic Studies Conducted in Populations of African Descent

Africa is a region of considerable genetic, cultural, geographical and phenotypic diversity [47,48]. These variable factors among Africans, especially genetic, environmental and dietary are critical for understanding the genetic risk factors for disease, gene-environment interactions and epigenetic basis of human diseases [47]. However, most studies have focused on non-African residents; hence, the interplay between these factors and human genetic diseases are scanty. Studying the epigenome is important, as alterations in epigenetic processes drive many diseases, including cancer [31].

Ethnic disparities in cancer measures such as incidence, prevalence, mortality, survival and morbidity have been reported. For instance, increased risk and mortality of prostate, cervical and breast and other cancers have been observed among people of African descent compared with those of Caucasian origin [49]. Although the cause of these disparities remains obscure, the high genetic diversity in Africa and/or epigenetic modifications could be contributing factors. However, while studies on the epigenetic basis of different types of cancers, such as prostate, cervical, endometrial, thyroid, breast, colorectal and lung cancer, among others, have mainly focused on mostly Caucasians and African Americans to a lesser extent [50] as summarized in Table 1, cancer epigenetics studies on people residing in Africa are largely lacking.

Africans also have a different diet, lifestyle and an environment that harbors many infectious pathogens capable of driving cancer [59]. Hence, it is likely that African populations have both genetic and environmental influences on the methylome [50]. Consequently, resident Africans are more exposed to pathogenic infections compared to the non-resident Africans. However, knowledge on the role of epigenetics in cancer in Africa is usually drawn from studies conducted among African Americans, which should not be extrapolated to native Africans due to admixture [49,60] and the higher genetic diversity in extant Africans. Epigenetic therapy is a rapidly advancing field of cancer research, since targeting epigenetic aberrations offer remarkable promise as a potential anti-cancer therapy, given the reversible nature of epigenetic changes [61]. Therefore, the lack of cancer epigenetics studies in Africa might have negative implications for people residing in Africa, as treatment development from studies in other ethnicities might not be effective for people of African descent. This calls for African-tailored cancer-related epigenetic studies to inspire African-specific cancer diagnostics and therapies. Advances in scientific research approaches, such as sequencing technologies, enabling the characterization of tumor phenotypes on a large scale, have highlighted epigenetic changes as a hallmark of cancer [61] and are promising solutions to overcome longstanding limitations in cancer epigenetics research [62]. While sequencing technologies are being applied in cancer epigenetic research in other parts of the world, their usage in Africa is relatively low, partly due to limited resources and technical know-how [63]. Therefore, more initiatives such as the Human Heredity and Health in Africa (H3Africa) consortium, a platform for collaboration and capacity building, should be created to focus on cancer epigenetics in Africa. Such initiatives will be invaluable in generating funds, training younger researchers and capacity building in cancer epigenetics research and development of cancer diagnostics and therapeutics.

## 4. Correlating the Prevalence of Tropical Pathogens and Cancer Prevalence

Besides causing infectious diseases, the role of infectious agents in non-communicable diseases including cancers is increasingly being appreciated. Currently, infections collectively account for 25–50% of all human cancers [64].

Infectious carcinogens affecting humans span all classes of microbial pathogens—viruses, bacteria, fungi and parasites. Among viruses, the Epstein-Barr virus (EBV), hepatitis C virus (HCV), hepatitis B virus (HBV), Kaposi’s sarcoma herpesvirus (KSHV), human immunodeficiency virus type-1 (HIV-1), human T cell lymphotropic virus type-1 (HTLV-1) and high-risk HPV (HR-HPV) genotypes are well-linked to cancer [65]. Notably, HR-HPV, HBV and HCV accounted for 690,000, 360,000 and 160,000 new cancer cases worldwide, respectively, in 2018 [3]. Most of these viral pathogens have been reported in Africa. For instance, the prevalence of HBV and HCV among the general population in the World Health Organization’s African Region is estimated at 7.5% and 1.0%, respectively, while for HIV, the prevalence is 3.6% among adults [66,67]. EBV is the most ubiquitous infectious pathogen, infecting up to 90% of the world’s population (reviewed in [68]). EBV is associated with various cancers of epithelial or hematopoietic cell origin worldwide. In Africa, the virus has been associated with endemic Burkitt’s lymphoma and other cancers such as nasopharyngeal carcinoma [69].

There are relatively fewer bacteria known to cause cancer. Thus far, *Helicobacter pylori,* a gastric ulcer-associated pathogen, is the only bacterium that has been clearly shown to be oncogenic. *H. pylori* causes both gastric cardia and gastric non-cardia adenocarcinoma [70], as well as non-Hodgkin lymphoma of gastric location [71]. Globally, *H. pylori* was the leading cause of infection-related cancer in 2018, being responsible for 810,000 new cancer cases [3]. Limited epidemiological evidence indicates a sexually transmitted bacterium, *Chlamydia trachomatis*, in ovarian cancer [72] and as a cofactor of HPV-associated cervical cancer [73]. This evidence is further supported by the extensive host DNA damage and depletion of the tumor suppressor p53 observed during *Chlamydia* infection [74]. Furthermore, a nationwide cohort study in Taiwan has associated genitourinary tuberculosis with urothelial cancer [75]. Together, these observations underscore the need for more studies to explore the role of bacteria in human cancers.

Three parasites, namely, *Schistosoma haematobium*, *Opisthorchis viverrini* and *Clonorchis sinensis*, are also considered oncogenic pathogens [76]. A link between *S. haematobium* and bladder cancer has long been established [77]. Globally, a total of 550,000 new cases of bladder cancer were reported in 2018 with *S. haematobium* accounting for about 1.1% [3]. The role of *S. haematobium* in bladder cancer is likely to be higher in Africa, where about 90% of those requiring treatment globally for schistosomiasis reside [78].

In addition to *S. haematobium,* three foodborne liver flukes, *Opisthorchis viverrini, Opisthorchis felineus* and *Clonorchis sinensis*, have been linked to hepatic cancers [79,80,81]. Although these parasites have not been reported in Africa, chronic infection by these parasites has been linked to cholangiocarcinoma, a bile duct cancer, in other parts of the world [82]. In 2018, *O. viverrini* and *C. sinensis* together accounted for 3500 (2.7%) of the 130,000 new cases of cholangiocarcinoma reported globally [3].

*Aspergillus flavus*, a fungus that is more prevalent in tropical regions, produces mycotoxins, particularly B1 aflatoxins, which are known to cause hepatocellular carcinoma (HCC) [83,84], as well as lung cancers [85]. Similarly, two other mycotoxins, ochratoxin and sterigmatocystin produced by fungi of the genus *Aspergillus*, have been implicated in some malignancies such as breast and testicular cancers [86]. In addition, a recent study by Aykut et al. demonstrated that in animal models and humans, mycological dysbiosis of the gut favoring infiltration of the pancreas by *Malassezia* species promotes pancreatic ductal adenocarcinoma [87]. Collectively, these findings highlight the role of fungi or their metabolites in many malignancies.

The involvement of infectious pathogens in carcinogenesis poses a serious threat to cancer prevention, especially in tropical climates, where the burden of infection is high. Unfortunately, many aspects of this involvement such as the mechanism of infection-induced carcinogenesis, have not been elucidated. Hence, further studies are needed to unravel the links between infection and cancers to help accelerate diagnostics, drug and vaccine development.

## 5. Mechanisms by which Pathogens Induce Cancer-Associated Epigenetic Alterations

Parasites hijack host cell signaling pathways to orchestrate epigenetic changes in the host. Studies have reported that parasitic infections result in extensive changes in gene expression profiles [88,89]. This remodeling of host genome activity is considered ‘epigenetic’ since it does not involve changes in the host DNA sequence but leads to stable and heritable gene expression profiles. Intracellular parasites also manipulate host gene expression directly by imprinting epigenetic marks on the host epigenome [89]. Furthermore, it has been demonstrated that parasite-induced signaling pathways can lead to stable changes in chromatin structure and epigenetic mechanisms that alter cellular phenotypes [89]. Moreover, intracellular parasites could be employing two strategies to initiate host signaling events. There might be direct secretion of parasite-derived proteins into the host cell cytoplasm, which could then interact with host proteins and affect their functions. On the other hand, parasites could be relying on a parallel strategy which involves sequestration of host proteins, leading to host signaling, which alters the chromatin structure [89].

The mechanisms by which pathogens induce epigenetic modifications vary among the various microbial pathogen classes such as bacteria, viruses, parasites and fungi. First, aberrant DNA methylation may be induced by the direct action of a pathogen or indirectly via chronic inflammation associated with infection [90,91]. For instance, the hepatitis B virus protein X (HBx) is known to directly induce aberrant DNA methylation. The HBx protein induces DNA methyltransferase (DNMT) upregulation, leading to DNA methylation of genes involved in the Ras pathway and angiogenesis [92]. In addition, EBV infection is considered to induce aberrant DNA methylation directly via the dysregulation of DNMTs. Notably, the aberrant DNA methylation first accumulates in non-cancerous or pre-cancerous tissues, leading to an ‘epigenetic field for cancerization’ that is highly predisposed to cancer development [93]. Infectious pathogens also induce chronic inflammation leading to aberrant DNA methylation [7] as shown for *Helicobacter pylori* and hepatitis viruses [91,94].

Pathogens also induce epigenetic changes via histone modification. Oncogenic viruses latently infect cells and dysregulate histone deacetylases (HDACs), leading to the suppression of tumor suppressor genes, thereby predisposing these cells to tumorigenesis. Viruses potentially modulate HDACs in several ways. Firstly, viruses modulate the expression of HDAC at the transcriptional level [95,96]. Secondly, oncogenic viruses disrupt HDAC-containing complexes [97,98]. Lastly, the viruses could be modulating HDACs indirectly via upstream factors [99,100].

**Figure 2:**
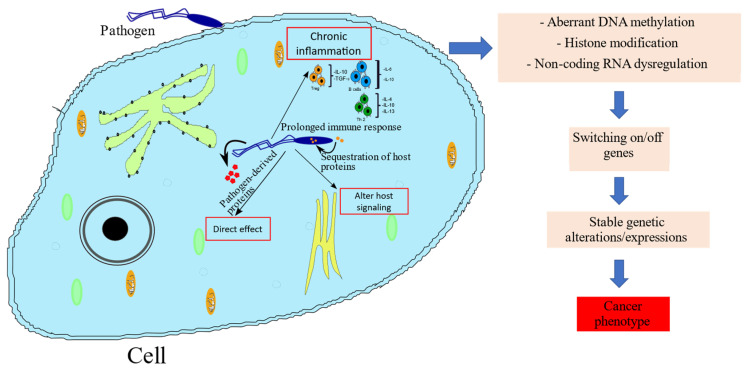
Schematic of mechanisms by which pathogens induce cancer-mediating epigenetic modifications. Upon entry into a cell, a pathogen may release pathogen-derived factors directly into the cytoplasm, alter host inflammatory responses and/or sequester host proteins. These events may lead to epigenetic changes, which stably alter the host genetic expression pattern, potentially leading to carcinogenesis.

Bacteria such as *H. pylori* potentially induce aberrant DNA methylation directly by triggering intracellular signaling, resulting in the activation of DNMTs being injected into gastric epithelial cells through a bacterial type IV secretion system [101] or indirectly owing to inflammation triggered by *H. pylori* infection. Notably, the inflammation resulting in aberrant DNA methylation results from *H. pylori* infection, and not from the direct activity of the bacterium [7]. More specifically, chronic inflammation may disrupt factors that protect DNA from methylation, such as ten-eleven translocation (TET) proteins via signals from macrophages or nitric oxide [7]. In addition, some Gram-positive anaerobic bacteria in the human colon produce butyrate (reviewed in [102]), which inhibits several classes of HDACs [103], potentially altering carcinogenesis pathways. Figure 2 summarizes the potential mechanisms employed by pathogens to drive epigenetic-based carcinogenesis.

The general involvement of epigenetics in host–pathogen interactions is likely to promote carcinogenesis. To keep up with the rapidly changing host environment, pathogens must change their gene expression patterns frequently. For instance, *Plasmodium* parasites have a complex vertebrate host life cycle, yet the parasite lacks most transcription factors [104]. However, these parasites are rich in histone variants, chromatin and histone-modifying enzymes [105,106], which enables them to survive through the changing host life cycle. It has been shown that *Mycobacterium tuberculosis* within the alveolar macrophages, for example inhibits interferon-γ-induced expression of several immune genes through histone acetylation [107], which explains the persistence of long-term chronic tuberculosis infections among some patients [108]. These examples and many more (reviewed in [108]) point towards the pathogen altering the host’s epigenetic factors, which could ultimately lead to cancer. More studies are needed to elucidate the underlying mechanisms.

## 6. Opportunities for Development of Treatment for Tropical Pathogen-Induced Cancers in Africa

Racial disparities in epigenetic patterns may indicate that Africans may differ from other races in terms of susceptibility and progression to diseases such as cancer [49,109,110]. Findings from epigenetic studies conducted in African American, Asian and Caucasian populations have been used to develop treatments and diagnostic techniques for cancer [111]. Although African Americans are of African descent, genetically they are not fully representative of Africans [112]. Furthermore, evidence indicates that lifestyle and environmental factors may also affect epigenetic changes, such as DNA methylation and histone modifications [113]. Therefore, cancer treatment and diagnostic techniques inspired by unadmixed African-based epigenetic studies are still lacking.

Biomarker discoveries have led to the design of early diagnostic, prognostic and treatment of different types of cancers [114]. Epigenetic diagnostic biomarkers have been widely discovered in other ethnicities and have been used in cancers such as prostate, breast, bladder and gastric cancer [115,116,117,118]. For example, Methylated *RASSF1A* serves as a diagnostic biomarker for African American populations in breast cancer [111] and a set of methylated genes (*glutathione S-transferase pi* gene (*GSTP1*), *RASSF1* and *APC*) have been used to develop the ConfirmMDx^®^ assay as a prognostic test in prostate cancer for European Americans [119]. However, there are no diagnostic techniques specific for African populations. Dedicated efforts are needed towards identifying unique epigenetic-based diagnostics for tropical pathogen-induced cancer.

Drugs that can reverse DNA methylation and histone modification in cancer have also been developed for other populations, such as Caucasians and Asians [31]. Currently, two classes of epigenetic drugs have been approved, namely DNA methylation inhibitors, which include 5-azacytidine and 5-aza-2′-deoxycytidine [31] and histone deacetylase inhibitors (HDACis) comprised of Zolinza and Istodax. Reports have shown that these drugs are effective against hematopoietic malignancies in Caucasian Americans [120]. However, the available epigenetic drugs are likely to have different effectiveness among people of African descent due to genetic differences that may result in drug metabolism differences [121]. Furthermore, the current FDA-approved epigenetic drugs are largely non-specific and may induce potentially lethal off-target effects [122]. Perhaps tailoring drugs to target specific pathogen-induced epigenetic changes could improve the selectivity and specificity of these drugs.

Evidently, epigenetics is important in modulating biological interactions between hosts and pathogens in pathogen-induced cancers. Therefore, prevention and treatment strategies aimed at arresting the developmental switches of tropical pathogens within the host could be used to block their virulence, which is linked to their survival and transmission to the host [108]. This stage is also linked to the survival and transmission of the pathogen to the host and may provide novel targets for epigenetic therapy [108]. Novel drugs targeting epigenetic changes induced by pathogenic organisms could be a better approach to drug development. These drugs will exhibit selectivity to the specific pathogen-induced epigenetic modifications and are less likely to potentiate drug resistance development due to lower selective pressure [123]. A long-term approach to reducing pathogen-induced cancers in African countries is by prevention through vaccination against infectious pathogens common in these settings [124]. This approach is likely to be more effective and economical than the non-specific epigenetic drugs, considering the endemicity of pathogens in these countries.

## 7. Conclusions

Despite the steady surge of cancers in Africa, significant progress in curbing the disease is still far from realization. Whereas epigenetic studies are a promising avenue for cancer detection and management, there is a significant paucity of data from the African continent. Infectious pathogens mediate epigenetic alterations associated with carcinogenesis, and Africa carries the highest burden of infections. This suggests a predisposition to pathogen-induced epigenetic changes that may lead to cancer development among native Africans. Thus, detailed studies on the patterns of epigenetic deregulation, as well as the role of tropical pathogens in infection-associated cancer in Africa are crucial. Such studies are likely to identify targets for African-specific prevention, diagnosis and therapeutic strategies that could ultimately tame the rising tide of cancer-related cases and mortalities in Africa.

## Figures and Tables

**Table 1:** Epigenetic modifications in African Americans and the genes implicated.

Type of Cancer	Epigenetic Modification	Genes/miRNA Involved	Differences between African Americans and Caucasians	Link with Disease Progression	References
Prostate	Hypermethylation	*CD44*, *GSTP1*	Higher frequency of CD44 hypermethylation in African Americans	Not indicated	[51]
	Hypermethylation	*Nkx2-5*, *TIMP3 AR*, *RARβ2*, *SPARC*	Higher methylation in African Americans	Yes	[52]
	Hypermethylated	*miR-152*	Higher methylation in African Americans	Yes	[53]
Colorectal	Hypermethylation	*CHL1*, *NELL1*, *GDF1*, *ARHGEF4*, *ITGA4*	Higher methylation in African Americans	Not indicated	[54]
	Hypermethylation	*miR-9*, *miR-124*, *miR-137*, *miR-548*, *miR-663*, *miR-2682*, *miR-6130*	Higher methylation in African Americans	Not indicated	[54]
Breast	Hypermethylation	*CDH13*, *HIN-1*, *TWIST*, *RAR-β*, *RASSF1A*	Higher methylation in African Americans	Not indicated	[55]
Thyroid	miRNA Upregulation	*miR-31*, *miR-221*	Upregulated in Caucasians downregulated in African Americans	Yes	[56]
Endometrial	RNA Down-regulation	*miR-337*	Downregulated in Caucasians compared with African Americans	Not indicated	[57]
Lung	Hypomethylation	*SHISA4*, *RAD1*	Decreased DNA methylation in African Americans	Yes	[58]
